# Cellular hnRNP A1 Interacts with Nucleocapsid Protein of Porcine Epidemic Diarrhea Virus and Impairs Viral Replication

**DOI:** 10.3390/v10030127

**Published:** 2018-03-13

**Authors:** Zhonghua Li, Wei Zeng, Shiyi Ye, Jian Lv, Axiu Nie, Bingzhou Zhang, Yumei Sun, Heyou Han, Qigai He

**Affiliations:** 1 State Key Laboratory of Agricultural Microbiology, College of Veterinary Medicine, Huazhong Agricultural University, Wuhan 430070, China; lzh1990@webmail.hzau.edu.cn (Z.L.); aiyouwei0620@163.com (W.Z.); yeshiyi@webmail.hzau.edu.cn (S.Y.); abing0313@webmail.hzau.edu.cn (B.Z.); sym@webmail.hzau.edu.cn (Y.S.); 2 State Key Laboratory of Agricultural Microbiology, College of Science, Huazhong Agricultural University, Wuhan 430070, China; lvjian1994@webmail.hzau.edu.cn (J.L.); nieaxiu00@163.com (A.N.); hyhan@mail.hzau.edu.cn (H.H.)

**Keywords:** nucleocapsid protein, porcine epidemic diarrhea virus (PEDV), heterogeneous nuclear ribonucleoprotein A1 (hnRNP A1)

## Abstract

The nucleocapsid (N) protein is a major structural component of porcine epidemic diarrhea virus (PEDV), which is predicted to be a multifunctional protein in viral replication. Heterogeneous nuclear ribonucleoprotein A1 (hnRNP A1) is a cellular protein participating in the splicing of pre-mRNA in the nucleus and translation regulation in the cytoplasm. According to our previous proteomic study about PEDV infection in vivo, hnRNP A1 was thought to be a cellular factor influencing PEDV replication. In this report, PEDV N protein was discovered to colocalize with cellular hnRNP A1 in perinuclear region of PEDV infected cells. Co-immunoprecipitation (CO-IP) results clearly demonstrated that PEDV N protein could bind to human hnRNP A1. Replication of PEDV was inhibited by silencing the expression of hnRNP A1 in CCL-81 cells, suggesting the positive effect of hnRNP A1 on PEDV infection.

## 1. Introduction

Porcine epidemic diarrhea virus is the most important viral agent which can cause diarrhea in pigs. Serious threat has been posed on the world pig industry as a result of the high morbidity and mortality caused by PEDV in piglets [1,2,3,4,5,6,7]. PEDV, a single-stranded positive sense RNA virus, is a member of coronavirus. Four structural proteins, including spike protein (S), membrane protein (M), envelope protein (E) and nucleocapsid protein (N), and the genome constitute the virion.

PEDV N protein has multiple functions. Firstly, as a structural protein, N Protein along with the genomic RNA forms the nucleocapsid of PEDV. Furthermore, N protein plays an important role in PEDV RNA synthesis and enhancing the PEDV transcription and virion assembly [8,9,10]. However, the effects of N protein on PEDV infection usually depend on its interaction with some host factors. For example, PEDV N protein binds with phosphoprotein nucleophosmin (NPM1) and then protects it from proteolytic degradation by caspase-3, enhancing cell survival and PEDV growth [11]. In addition, it has been proved that interaction between PEDV N protein and TBK1 could inhibit IRF3 activation and type I IFN production, further to causing the circumvention of the host’s antiviral immunity [12].

More than 20 heterogeneous nuclear ribonucleoproteins (hnRNPs) have been discovered and among them hnRNP A1 is the best-characterized one [13,14]. HnRNP A1 is a RNA-binding protein which functions as binding to pre-mRNA to form hnRNP particles in eukaryotic cells [15]. This protein contains two RNA-binding domains (RBDs) and a glycine-rich domain responsible for protein-protein interaction [16,17]. It has been reported that hnRNP A1 selectively interacts with different RNA-binding proteins through its glycine-rich domain [18]. Previous studies have demonstrated hnRNP A1 could interact with N proteins of SARS Coronavirus and mouse hepatitis virus (MHV) [14,19]. Since PEDV is also a member of coronavirus and PEDV N protein is a RNA binding protein, it is hypothesized that PEDV N protein might also be able to interact with hnRNP A1 during PEDV infection.

Our previous work has proved that hnRNP A1 underwent different regulations in jejunum tissues of piglets infected with PEDV virulent strain and its attenuated strain [20]. Therefore, we assume that hnRNP A1 may play a role in the life cycle of PEDV. In this study, it is found that hnRNP A1 could interact with PEDV N protein and PEDV replication could be inhibited by silencing the hnRNP A1.

## 2. Materials and Methods

### 2.1. Virus, Cells, Antibodies, and Plasmid

The PEDV YN144 (GenBank accession no. KT021232), YN13 (GenBank accession no. KT021228), and CV777 (GenBank accession no. AF353511.1) strain were used throughout this study. YN13 and YN144 strains were obtained by passaging the YN strain, a variant strain isolated from the intestine of a piglet with diarrhea, for 13 and 144 generations, respectively. Our previous study has demonstrated that YN13 was a virulent strain and YN144 was an attenuation strain [20]. CV777 strain, a classical PEDV strain, was provided by Chengdu Tecbond Biological Product Co., Ltd. (Chengdu, China).

The CCL-81 cell line and HEK293T cell line were purchased from American Type Culture Collection (ATCC) and cultured in Dulbecco’s modified Eagle’s medium (DMEM), supplemented with 10% fetal bovine serum (Invitrogen, Carlsbad, CA, USA) at 37 °C with 5% CO_2_. The CCL-81 cell line was used for virus growth, infection, and cell lysate preparation. HEK293T cell line was used for CO-IP analysis.

The rabbit anti-hnRNP A1, anti-flag polyclonal antibody (PAb) and mouse anti-β-actin, anti-flag mono-antibody (MAb) were purchased from ABclonal (Wuhan, China). Mouse MAb against PEDV N protein was purchased from Youlong Biotech (Shanghai, China). Mouse MAb against PEDV spike protein was established by our laboratory. Application of this MAb has been described in some previous studies [21,22]. Alexa Fluor 488-conjugated anti-rabbit, anti-mouse and Alexa Fluor 594-conjugated anti-mouse antibodies were purchased from AntGene Biological (Wuhan, China).

The cDNA expression construct encoding YN144 N protein was PCR amplified and cloned into pCAGGS-FlagC, which encode a C-terminal flag.

### 2.2. Animal Experiment and Proteome Study

Details of this part have been described in our previous study [20]. Briefly, twelve piglets were randomly divided into three groups of four. The piglets in different groups were orally administrated with 4.22 mL YN13 and 4.22 mL YN144 with the same titer of 10^5.375^ Median tissue culture infective dose (TCID_50_) mL^−1^ and 4.22 mL DMEM, respectively. All piglets were euthanized and necropsied when diarrhea was observed in the piglets in YN13-infected group. Jejunum tissues were separated rapidly, washed with ice-cold PBS buffer, snap-frozen in liquid nitrogen, and kept at −80 °C for subsequent proteome study. For the proteome study, iTRAQ labeling coupled with LC-MS/MS was chose to analyze whole cell changes of jejunum of piglets, infected with PEDV YN13 strain and YN144 strain.

### 2.3. Immunoprecipitation and Immunoblotting

HEK293T cells were transfected with the vector described above. Cells were harvested at 24 h post transfection (hpt), washed three times with cold PBS (pH 7.4), lysed with IP lysis buffer (Beyotime, Shanghai, China) containing 1 mM phenylmethylsulfonyl fluoride (PMSF) at 4 °C. Clarified extracts were precleared with protein A/G agarose (Beyotime, Shanghai, China) for 2 h then centrifuged at 3000 rpm for 5 min at 4 °C. Supernatants were incubated with mono-antibody against Flag for 12 h, then protein A/G beads were added and incubated at 4 °C for 6 h. The beads were then washed with IP lysis buffer five times and boiled in sample buffer, and the proteins were subjected to SDS-PAGE, followed by immunoblotting analysis with anti-Flag PAb or anti-hnRNP A1 PAb.

### 2.4. Immunofluorescence and Confocal Microscopy

CCL-81 cells grown on coverslips were infected with PEDV YN144 strain, YN13 strain and CV777 strain, respectively, at a multiplicity of infection (MOI) 0.001. At 12 h post infection (hpi), the cells were fixed with 4% paraformaldehyde for 10 min followed by being treated with methanol. Fixed cells were blocked with 5% bovine serum albumin and incubated with anti-hnRNP A1 PAb and MAb against PEDV N protein. Alexa Fluor 488-conjugated anti-rabbit and Alexa Fluor 594-conjugated anti-mouse antibodies were served as the secondary antibody. Cell nucleus were stained with 4′,6-diamidino-2-phenylindole (DAPI). The localization of PEDV N protein, hnRNP A1 and cell nucleus were then observed on a ZEISS confocal microscope (ZEISS, Oberkochen, Germany).

### 2.5. RNA Interference

CCL-81 cells were transfected with siRNAs targeting to hnRNP A1 with Lipofectamine 2000 reagent (Invitrogen, Carlsbad, CA, USA) according to the manufacturer’s instructions. The siRNAs targeting hnRNP A1 were synthesized by Gene Pharma (Shanghai, China). Effect of RNA interference was tested by Western blot at 60 hpt.

### 2.6. Indirect Immunofluorescence Assay

CCL-81 cells seeded in a 24-well plate were transfected without SiRNA or with 20 nM nonspecial control SiRNA or SiRNA1 targeting to hnRNP A1. At 60 hpt, cells were infected with or without PEDV. At 12 hpi (YN144) or 24 hpi (YN13 and CV777), the cells were fixed with 4% paraformaldehyde followed by treating with methanol. Then the fixed cell was blocked with 5% bovine serum albumin and then incubated with MAb against PEDV S protein. Alexa Fluor 488-conjugated anti-mouse antibody was applied to detect the primary antibodies. DAPI was selected to stain the cell nucleus.

### 2.7. Real-Time PCR

RNA was extracted by using Tripure Isolation Reagent (Roche, IN, USA) following the manufacturer’s instructions. The cDNA was obtained by RT-PCR using the Primescript™ RT Master mix (Takara, Tokyo, Japan). The real-time RT-PCR assay for quantifying PEDV genome used the following primer and probe sequences: PEDV forward primer: 5′-CGTACAGGTAAGTCAATTAC-3′, PEDV reverse primer: 5′-GATGAAGCATTGACTGAA-3′, PEDV Taq-Man^®^probe: FAM-TTCGTCACAGTCGCCAAGG-TAMRA.

### 2.8. Western Blot

Cells were lysed with lysis buffer (Beyotime, Shanghai, China) containing 1 mM PMSF. These proteins were subjected to SDS-PAGE and then the separated protein bands were transferred onto PVDF membrane using a trans-blot (Bio-Rad, Berkeley, CA, USA). The membrane was incubated in blocking buffer (Tris-buffered saline (TBS), containing 0.05% Tween-20 (TBST) and 5% skim milk) for 2 h at room temperature followed by washing three times by PBST. Then the membrane was incubated with the corresponding primary antibodies for 2 h at room temperature. After being washed three times by PBST, the membrane was incubated with (HRP)-conjugated goat-anti mouse/rabbit IgG at room temperature for 1.5 h. Finally, the protein bands were visualized using the Clarity™ Western ECL Blotting Substrate (Bio-Rad, Hercules, CA, USA).

## 3. Result

### 3.1. Different Regulations of hnRNP A1 in Jejunum Tissues Infected with PEDV YN13 Strain and YN144 Strain

**Figure 1:**
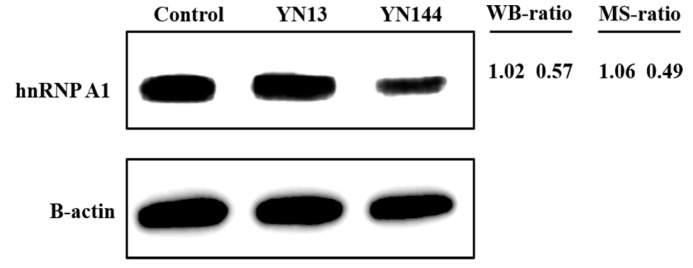
Confirmation of expression of hnRNP A1 by western blot. Analysis of hnRNP A1 expression level in the jejunum tissues of the control, YN13-infected and YN144-infected groups. The WB-ratio (western blotting ratio, PEDV infected/mock) was calculated based on the relative intensity of each band in PEDV-infected group to the corresponding band in control. β-actin was used as a loading control. The MS-ratio (infection/control) obtained by MS analysis are shown on the right.

According to the result of LC-MS/MS, hnRNP A1 was down-regulated in an attenuated PEDV strain (YN144) infected jejunum tissues, while showed no apparent change in a virulent PEDV strain (YN13) infected jejunum tissues [20]. Western blot assay was applied to confirm the changes of hnRNP A1. As shown in Figure 1, regulations of hnRNP A1 were consistent with the result of LC-MS/MS. HnRNP A1 has been proved to be involved in the replication process of other coronaviruses [14,23]. Therefore, we assumed that hnRNP A1 may play a role in PEDV replication and influence the pathogenicity of PEDV in vivo.

### 3.2. PEDV N Protein Colocalizes with Cellular hnRNP A1 Protein during PEDV Infection

**Figure 2:**
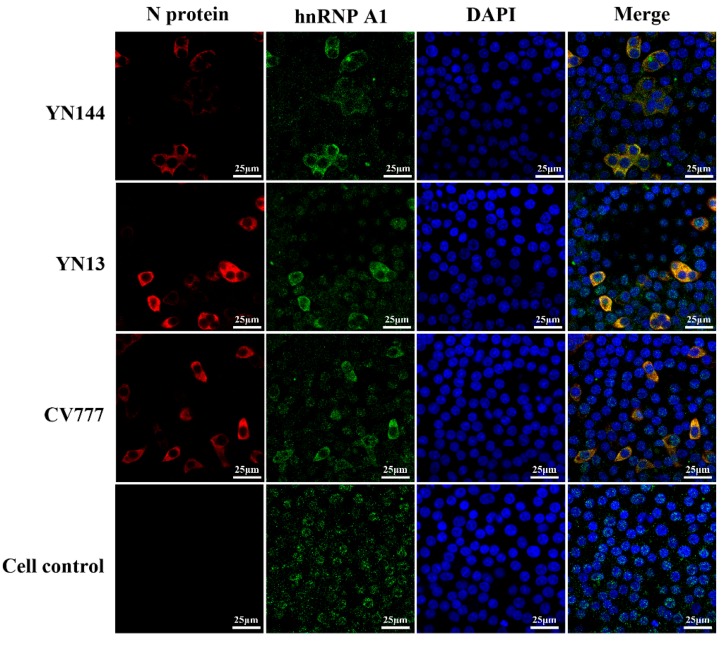
Co-localization of N protein with hnRNP A1. CCL-81 cells were infected with different PEDV strains. At 12 hpi, the localization of PEDV N protein and hnRNP A1 in cells was examined by immunofluorescence staining with anti-PEDV N protein and anti-hnRNP A1 antibodies. The nucleus is indicated by DAPI (blue) staining. PEDV N protein is indicated in green and the hnRNP A1 in red.

Previous studies have demonstrated that N protein of some coronaviruses, such as MHV and SARS-CoV, could interact with hnRNP A1. In this study, we determined to investigate whether N protein can be colocalized with hnRNP A1 during PEDV infection. CCL-81 cells were infected with the YN144, YN13, and CV777 strain of PEDV, respectively, and the localization of NP and hnRNP A1 was observed by confocal microscopy at 12 hpi. As shown in Figure 2, hnRNP A1 was mainly localized in the nucleus without PEDV infection. However, some hnRNP A1 was transported from the nucleus to the cytoplasm and colocalized with PEDV N protein during PEDV infection. Furthermore, PEDV N protein was localized in cytoplasm and no differences were found in the localization of N protein of these PEDV strains. The immunofluorescence assay demonstrated that PEDV N protein and hnRNP A1 colocalized in cytoplasm.

### 3.3. PEDV N Protein Interacts with Cellular hnRNP A1 Protein

**Figure 3:**
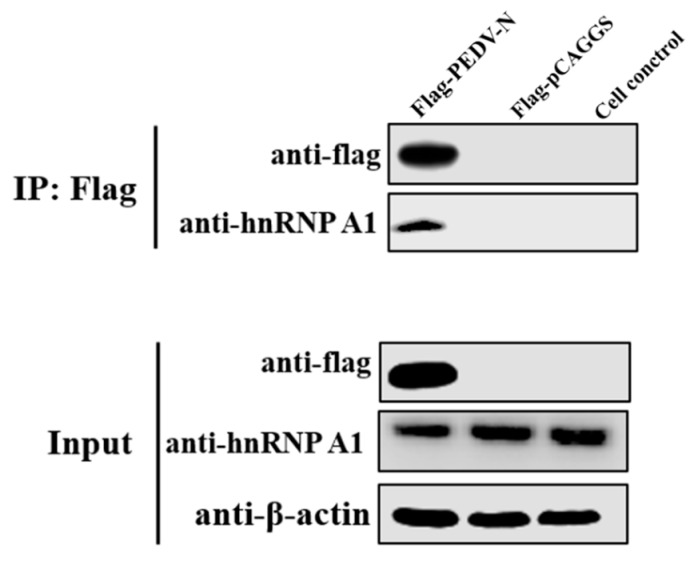
Interaction of hnRNP A1 and PEDV N protein. HEK293T cells were transfected with a vector expressing PEDV N protein with a flag tag (flag-PEDV-N) or empty vectors (flag-pCAGGS) and the whole-cell lysates obtained at 24 hpt were immunoprecipitated with anti-Flag mAb. After separation by SDS-PAGE, proteins were detected by immunoblotting with the indicated antibodies (IP: Flag). Cell lysates were also applied to confirm the expression of proteins (Input).

The colocalization of PEDV N protein and hnRNP A1 demonstrated that a certain interaction may exist between these two proteins. Thus, co-immunoprecipitation (CO-IP) was performed to testify this phenomenon. 293T cells transfected with plasmids expressing the flag-tagged PEDV N protein were subjected to immunoprecipitation using anti-flag antibody. Interaction of PEDV N protein with host protein hnRNP A1 was analyzed by immunoblotting with anti-flag antibody and anti-hnRNP A1 antibody. As shown in Figure 3, cellular hnRNP A1 protein was only detected in the presence of flag tagged PEDV N by CO-IP. These results indicate that N protein interacted with hnRNP A1.

### 3.4. Knockdown of hnRNP A1 Expression Inhibits YN144 Replication

**Figure 4:**
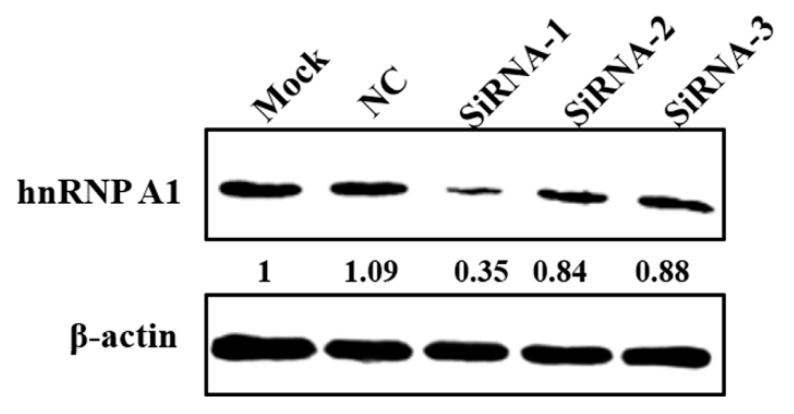
Silencing hnRNP A1 by siRNAs. CCL81 cells were transfected with no siRNA (Mock), negative control siRNA (NC), SiRNA-1, SiRNA-2 or SiRNA-3 at a concentration of 20 nM. Cells were collected at 60 hpt to analyze the expression of hnRNP A1 by western blot. HnRNP A1 protein levels were quantified by measuring band intensities and normalized with respect to the amount of β-actin.

In order to investigate whether hnRNP A1 participates in the replication of PEDV, three siRNAs targeting to hnRNP A1 were synthesized to silence the expression of this protein. Sequences of the siRNAs were shown in Table 1. CCL-81 cells were transfected with siRNAs against hnRNP A1 expression, cells were collected at 60 hpt to analyze the silencing efficiency of hnRNP A1 at the protein level. As shown in Figure 4, siRNA-1 showed the best performance for silencing hnRNP A1 and was chosen for the following research.

**Figure 5:**
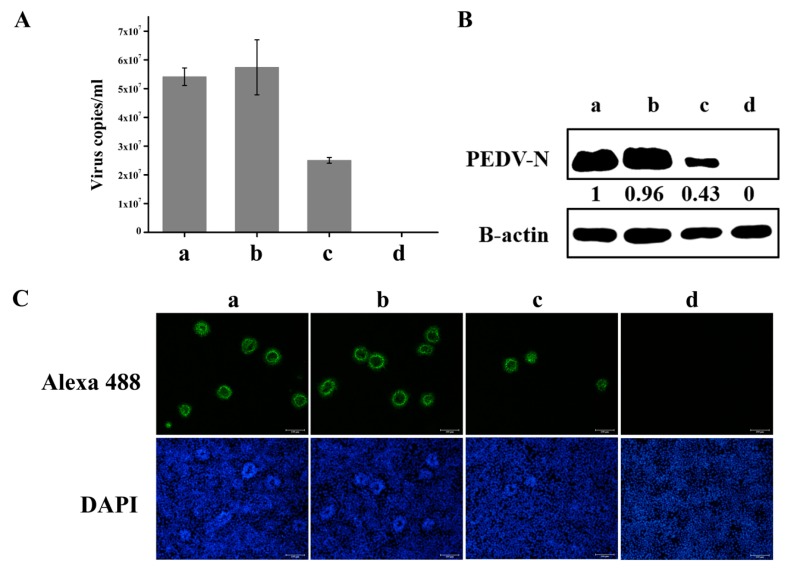
Knockdown of hnRNP A1 expression inhibits YN144 replication. CCL-81 cells were transfected without siRNA (a) or with NC (b) or siRNA-1 (c). At 60 hpt, cells were infected with YN144 Strain at a moi of 0.001 or mock infected (d). Virus suspensions were harvested at 12 hpi to calculate the virus titer by real-time RT-PCR. The virus copies per mL suspension was calculated (**A**). Cells were harvested at 12 hpi to analyze the expression of PEDV N protein by Western blot (**B**) and the numbers of cells infected with PEDV by IFA (Original magnification 100×) (**C**). PEDV N protein levels were quantified by measuring band intensities and normalized with respect to the amount of β-actin. Data are shown as means ± the standard errors of the mean (SEM) of at least three independent experiments, with the error bars representing the standard deviations.

CCL-81 cells were transfected with siRNA-1 for 60 h followed by infection with PEDV YN144 strain. These cells were subjected to western blot, indirect immunofluorescence assay (IFA) or real-time PCR at 12 hpi Figure 5. According to the results, a conclusion was reached that knockdown of hnRNP A1 reduced the replication of YN144.

### 3.5. Knockdown of hnRNP A1 Expression Inhibits YN13 and CV777 Replication

**Figure 6:**
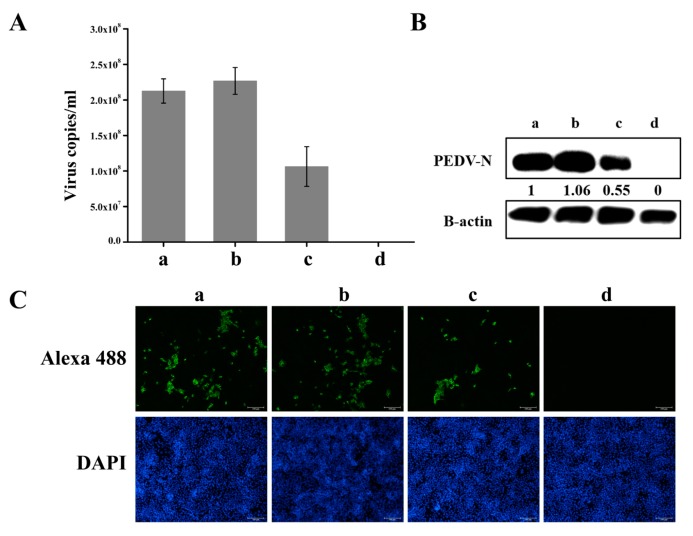
Knockdown of hnRNP A1 expression inhibits YN13 replication. CCL-81 cells were transfected without siRNA (a) or with NC (b) or siRNA-1 (c). At 60 hpt, cells were infected with YN13 Strain at a moi of 0.001 or mock infected (d). Virus suspensions were harvested at 24 hpi to calculate the virus titer by real-time RT-PCR. The virus copies per mL suspension was calculated (**A**). Cells were harvested at 24 hpi to analyze the expression of PEDV N protein by Western blot (**B**) and the numbers of cells infected with PEDV by IFA (Original magnification 100×) (**C**). PEDV N protein levels were quantified by measuring band intensities and normalized with respect to the amount of β-actin. Data are shown as means ± SEM of at least three independent experiments, with the error bars representing the standard deviations.

**Figure 7:**
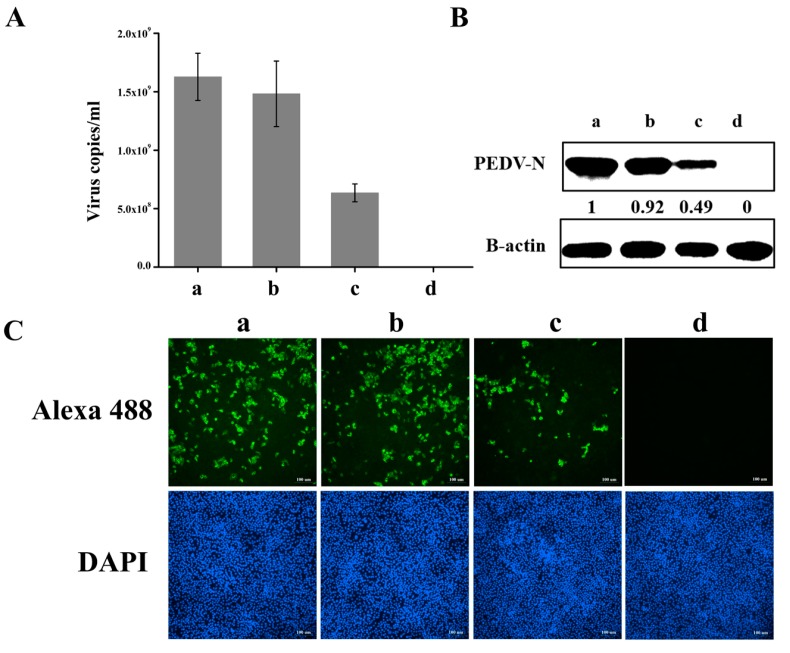
Knockdown of hnRNP A1 expression inhibits CV777 replication. CCL-81 cells were transfected without siRNA (a) or with NC (b) or siRNA-1 (c). At 60 hpt, cells were infected with CV777 Strain at a moi of 0.001 or mock infected (d). Virus suspensions were harvested at 24 hpi to calculate the virus titer by real-time RT-PCR. The virus copies per mL suspension was calculated (**A**). Cells were harvested at 24 hpi to analyze the expression of PEDV N protein by western blot (**B**) and the numbers of cells infected with PEDV by IFA (Original magnification 100×) (**C**). PEDV N protein levels were quantified by measuring band intensities and normalized with respect to the amount of β-actin. Data are shown as means ± the SEM of at least three independent experiments, with the error bars representing the standard deviations.

The PEDV virulent strain YN13 and PEDV classical strain CV777 were further applied to test the positive effects of hnRNP A1 on PEDV replication. As shown in Figure 6 and Figure 7, similar results were obtained. These results demonstrated that hnRNP A1 is a positive regulator of PEDV replication.

## 4. Discussion

In this study, interaction between hnRNP A1 and PEDV N protein was verified by CO-IP and immunofluorescence assay. Our results established that N protein interacts with hnRNP A1 in PEDV infected cells and 293T cells expressing PEDV N protein.

hnRNP A1 not only mainly localizes in the cell nucleus, but also shuttles between the nucleus and the cytoplasm [24,25]. However, this protein underwent a relocalization to cytoplasm during PEDV infection, suggesting a possible functional link between hnRNP A1 and PEDV infection. This phenomenon is very similar to a previous study about MHV [23]. Furthermore, we found that the PEDV N protein and hnRNP A1 co-localized predominantly in the perinuclear region of PEDV infected cells, in which active coronavirus replication/transcription complexes reside. This indicates both PEDV N protein and hnRNPA1 might participate in constituting the PEDV replication/transcription complex and their interaction may be involved in regulation of PEDV replication.

It is well known that hnRNP A1 is one part of replication/transcription complex of both SARS-CoV and MHV [8,14,23,26,27]. Overexpression of hnRNPA1 facilitates MHV replication while inhibition of hnRNP A1 expression results in the reduction of MHV replication [26]. Since PEDV is also a member of coronavirus, hnRNP A1 may be involved in the process of PEDV infection. In order to test this hypothesis, we silenced the hnRNP A1 and then studied its effect on PEDV infection. Similar to the result of MHV following silencing hnRNP A1, replication of three PEDV strains were all inhibited, suggesting a positive effect of hnRNP A1 on PEDV infection. Replication of coronavirus depend on the generation of nested subgenomic mRNAs (sgmRNAs) with a common capped 5′ leader sequence [28]. Optical transcription of sgmRNAs requires the interaction between its 5′ leader sequence and the intergenic (IG) sequences of each ORF. It has been reported that hnRNP A1 can bind to both the 5′ terminal leader sequences and IG sequences [29]. So, inhibition of PEDV by silencing hnRNP A1 is likely due to the break of the interaction between 5′ terminal leader sequences and IG sequences. However, till now we have not got evidence to support it. In addition, silencing of hnRNP A1 might also be able to reduce its interaction with PEDV N protein. The function of their interaction on PEDV infection is under the investigation of our laboratory.

Our previous study has demonstrated that hnRNP A1 was downregulated in the jejunum of PEDV strain YN144 infected group, but no apparent change in group infected with YN13 [20]. Since YN13 caused diarrhea in piglets while YN144 could not, therefore, downregulation of hnRNP A1 was one of the reasons responsible for the lower pathogenicity of YN144 than YN13 in vivo.

## Figures and Tables

**Table 1:** Sequences of siRNAs against hnRNP A1 expression.

Name	Sequences (5′-3′)
SiRNA-1	GGAAGAGUUGUGGAACCAATT
SiRNA-2	GGAUUUGGUAAUGAUGGAATT
SiRNA-3	GCGGUGGAGGUCAAUACUUTT
Negative control siRNA (NC)	UUCUCCGAACGUGUCACGUTT
